# Simple Renal Cysts and Aortic Aneurysms: From Imaging Markers to Prognostic Indicators of Postoperative Outcomes

**DOI:** 10.3400/avd.ra.26-00044

**Published:** 2026-09-03

**Authors:** Toshiya Nishibe, Masaki Kano, Shinobu Akiyama, Shoji Fukuda, Tomohiro Nakajima, Kenichi Kato, Masayasu Nishibe, Alan Dardik

**Affiliations:** 1 Department of Medical Informatics and Management, Hokkaido Information University, Ebetsu, Hokkaido, Japan; 2 Department of Cardiovascular Surgery, Tokyo Medical University, Tokyo, Japan; 3 Division of Cardiovascular Surgery, Department of Surgery, Sapporo Medical University, Sapporo, Hokkaido, Japan; 4 Department of Surgery, Eniwa Midorino Clinic, Eniwa, Hokkaido, Japan; 5 Department of Vascular Surgery, Icahn School of Medicine at Mount Sinai, New York, USA

**Keywords:** abdominal aortic aneurysm, thoracic aortic disease, simple renal cyst, endovascular aneurysm repair, thoracic endovascular aortic repair

## Abstract

Simple renal cysts (SRCs) have traditionally been regarded as benign, age-related incidental findings with limited clinical significance. However, accumulating evidence suggests that SRCs are associated with abdominal aortic aneurysm (AAA), thoracic aortic aneurysm (TAA), and aortic dissection (AD), indicating that they may reflect systemic extracellular matrix (ECM) degeneration rather than isolated renal aging. This narrative review summarizes the current evidence linking SRCs to aortic disease, explores the underlying biological mechanisms, and discusses their potential prognostic significance following endovascular aortic repair. Observational studies have consistently reported a 2- to 3-fold higher prevalence of SRCs in patients with aortic disease compared with controls. Shared mechanisms, including ECM remodeling, matrix metalloproteinase activation, genetic susceptibility, and arterial stiffening, provide biological plausibility for this association. Emerging evidence further suggests that SRCs are independently associated with reduced sac shrinkage after endovascular aneurysm repair (EVAR) and thoracic endovascular aortic repair (TEVAR), as well as with an increased risk of aortic-related adverse events following TEVAR for type B AD. Although the current evidence is derived predominantly from retrospective studies, SRCs represent a readily identifiable imaging marker that may enhance risk stratification and postoperative surveillance. Prospective multicenter studies are warranted to validate these findings and clarify their clinical implications.

## Introduction

Simple renal cysts (SRCs) are among the most frequently encountered incidental findings on abdominal imaging, particularly in aging populations. Traditionally regarded as benign lesions without clinical consequence, SRCs were long considered a routine byproduct of aging and localized structural changes without any meaningful systemic implications.^1)^ However, over the past 2 decades, this long-standing perception has undergone a significant shift. An expanding body of observational, imaging-based, and mechanistic research now supports the concept that SRCs may serve as imaging markers of systemic connective tissue degeneration, a biological vulnerability that also underlies abdominal aortic aneurysm (AAA), thoracic aortic aneurysm (TAA), and aortic dissection (AD). Extracellular matrix (ECM) weakening is fundamental to aortic wall failure, and the possibility that SRCs reflect a broader vascular susceptibility has attracted considerable attention.^2,3)^ Notably, enzymes involved in ECM remodeling, particularly matrix metalloproteinases (MMPs) such as MMP-2 and MMP-9, have been implicated in both renal cyst formation and aortic medial degeneration, suggesting a shared biochemical pathway that is explored in detail later in this review. The “guilt by association” paradigm further reinforces this interpretation, proposing that SRCs should be viewed not as isolated renal findings but as potential indicators of coexisting or subclinical vascular disease.^4)^

Several studies of endovascular aneurysm repair (EVAR) and thoracic endovascular aortic repair (TEVAR) have extended the significance of SRCs beyond their preoperative associations, demonstrating prognostic links with aneurysm sac shrinkage and long-term adverse events.^5–10)^ This dual role both as a marker of underlying aneurysm biology and as a prognostic marker of postoperative outcomes suggests that SRCs may enhance risk stratification across the continuum of care. This review synthesizes current evidence linking SRCs with aortic disease, explores putative biological mechanisms, and highlights the possible role of SRCs as a prognostic marker following EVAR and TEVAR. SRCs were defined as benign renal cystic lesions corresponding to Bosniak category I,^11)^ characterized by thin smooth walls, homogeneous fluid content, and no septa, calcification, solid components, or enhancement, which were consistent with the definitions used in the studies cited in this review.

## Literature Search Strategy (Narrative Review)

**Fig. 1:**
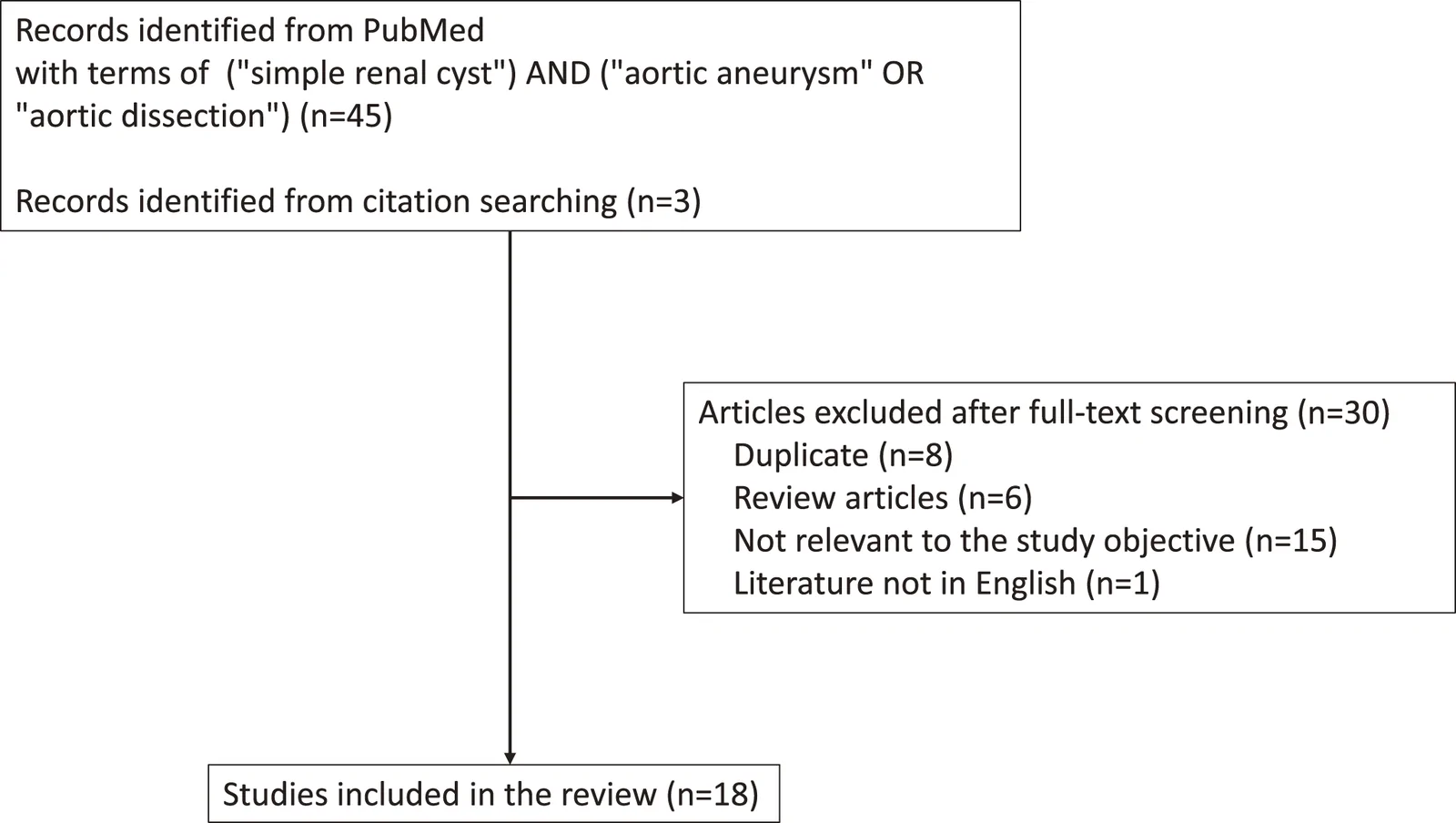
Flow diagram illustrating the identification, screening, and selection of studies investigating the association between simple renal cysts and aortic disease included in this narrative review.

A literature search of PubMed was conducted for articles published between January 2010 and July 2026 using the search strategy (“simple renal cyst”) AND (“aortic aneurysm” OR “aortic dissection”). Duplicate records were removed, followed by title and abstract screening. Irrelevant publications, letters, case reports, editorials, and conference abstracts were excluded. Additional eligible studies were identified through manual screening of the reference lists of relevant review articles and included studies (**Fig. 1**).

We explicitly did not perform a PRISMA (Preferred Reporting Items for Systematic Reviews and Meta-Analyses)-compliant systematic review^12)^; rather, we emphasize transparency regarding scope, including search principles and selection criteria in accordance with standards for high-quality narrative reviews. The review process followed SANRA guidelines (Scale for the Assessment of Narrative Review Articles) to enhance methodological transparency.^13)^ Several relevant studies identified through manual searching of reference lists were also included to provide a comprehensive overview of the topic.

## Clinical Association between SRCs and Aortic Disease

Accumulating evidence suggests that SRCs are associated with aortic disease. A large observational study of 35498 patients reported a higher prevalence of SRCs among patients with AAA, TAA, and AD than in control populations (AAA, 7.9% vs 3.3%; thoracic aortic disease, 10.1% vs 3.9%; ascending TAA, 8.0% vs 3.2%; descending TAA, 3.3% vs 0.9%; type A AD, 0.6% vs 0.2%; type B AD, 1.1% vs 0.3%) .^2)^ Conversely, a meta-analysis of 11 observational studies involving 19719 participants reported that the presence of SRCs was associated with increased odds of AAA (odds ratio [OR] 2.61), ascending TAA (OR 1.98), descending TAA (OR 3.44), type A AD (OR 1.98), and type B AD (OR 2.55).^3)^ These findings raise the possibility that SRCs may reflect systemic connective tissue degeneration and ECM fragility rather than merely incidental age-related lesions. The following subsections provide a detailed overview of the current evidence linking SRCs with AAA and thoracic aortic disease.

### SRCs as markers of AAA (Table 1)

**Table 1:** Simple renal cysts (SRCs) as markers of abdominal aortic aneurysm (AAA)

First author (Year)	Cases (AAA) (n)	Controls (n)	SRC in cases (%)	SRC in controls (%)	P-value	OR (95% CI)
Yaghoubian (2006)^14)^	100	100	54	30	<0.001*	NR
Ito (2010)^15)^	164	233	43.5	23.3	0.014*	NR
Pitoulias (2012)^16)^	110	60 (AOD)	69.1	26.7	<0.001*	NR
Gindera (2015)^18)^	236	236 (AOD)	68.6	37.3	0.002*	2.11 (1.33–3.36)
Spanos (2016)^19)^	100	100 (age-matched cohort)	NR	NR	<0.05*	NR
Veiga (2017)^20)^	91	396	41.8	37.4	0.473	NR
Song (2020)^17)^	271	1387	55	19	0.01*	NR
Miszczuk (2020)^21)^	97	100	74	57	<0.016*	NR
Nishibe (2022)^22)^	223	NR	59.2	NR	NR	NR

^*^Significant.

OR, odds ratio; CI, confidence interval; AOD, aortoiliac occlusive disease; NR, not reported

Multiple case–control studies using abdominal CT datasets have consistently shown a higher prevalence of SRCs in patients with AAA than in non-AAA populations, with SRCs reported in approximately one-third to more than half of AAA patients.^14–22)^ A meta-analysis from the ALICE group quantified this association, reporting a pooled OR of 2.54 (95% CI, 1.85–3.49) for SRC prevalence in AAA compared with controls.^23)^ Another meta-analysis pooling 11 studies confirmed increased odds of AAA in those with SRCs, with an OR of 2.61 (95% CI, 1.90–3.58), even after multivariable adjustment.^3)^ A more recent meta-analysis similarly demonstrated that patients with AAA exhibited an approximately 3-fold higher prevalence of SRCs, reinforcing a shared underlying pathophysiologic mechanism.^24)^ Although 1 case–control study by Veiga et al. reported a nonsignificant association, its limited sample size likely reduced statistical power.^20)^ However, the effect direction was consistent with other studies, and pooled analyses show that it does not materially influence the overall estimate.

Since abdominal CT is frequently performed for unrelated clinical indications, incidental detection of SRCs can serve as an opportunity to prompt careful evaluation of the abdominal aorta, particularly in older adults or individuals with vascular risk factors. Although SRCs alone are insufficient for a population-level screening tool, their presence increases the pretest probability of AAA and justifies meticulous review of aortic diameter on existing imaging.^4)^

### SRCs as markers of thoracic aortic disease (Table 2)

**Table 2:** Simple renal cysts (SRCs) and thoracic aortic disease (TAD)

First author (Year)	Disease focus	Cases (n)	Controls (n)	SRC in cases (%)	SRC in controls (%)	P-value	OR (95% CI)
Kim (2011)^25)^	AD	311	311	33.8	25.7	0.028	1.49 (1.12–1.98)
Ziganshin (2016)^26)^	TAD (Asc/Desc TAA; Type A/B AD)	842	543	42.5 (Asc 37.5/Desc 57.0/Type A 44.1/Type B 47.3)	15.3	<0.001	NR
Bouleti (2025)^27)^	AD within MFS	131 (MFS)	131 (matched cohort)	41	21	<0.001	2.30 (1.00–5.32)

OR, odds ratio; CI, confidence interval; AD, aortic dissection; Asc, ascending; desc, descending; TAA, thoracic aortic aneurysm; NR, not reported; MFS, Marfan syndrome

TAA is often clinically silent and challenging to detect before complications occur. Several studies suggest that SRCs may serve as actionable imaging markers for TAA.^25,26)^ In a single-center study of 842 patients with thoracic aortic disease, the prevalence of SRCs was 37.5% in ascending TAA, 57% in descending TAA, 44% in type A AD, and 47% in type B AD, compared with 15% in control subjects.^26)^ Subsequent syntheses recommended that SRCs should be considered among clinical cues that warrant dedicated thoracic aortic imaging in appropriate clinical settings described by Elefteriades et al.^4)^

Heritable connective tissue disorders further support this association. In Marfan syndrome, SRCs are more common than in age- and sex-matched controls, and multivariable analysis has shown that SRC presence is independently associated with AD.^27)^ These observations suggest that cyst formation may reflect generalized ECM fragility affecting both renal and thoracic aortic tissues.

## Biological and Pathophysiological Mechanisms Linking SRCs with Aortic Aneurysms (Fig. 2)

**Fig. 2:**
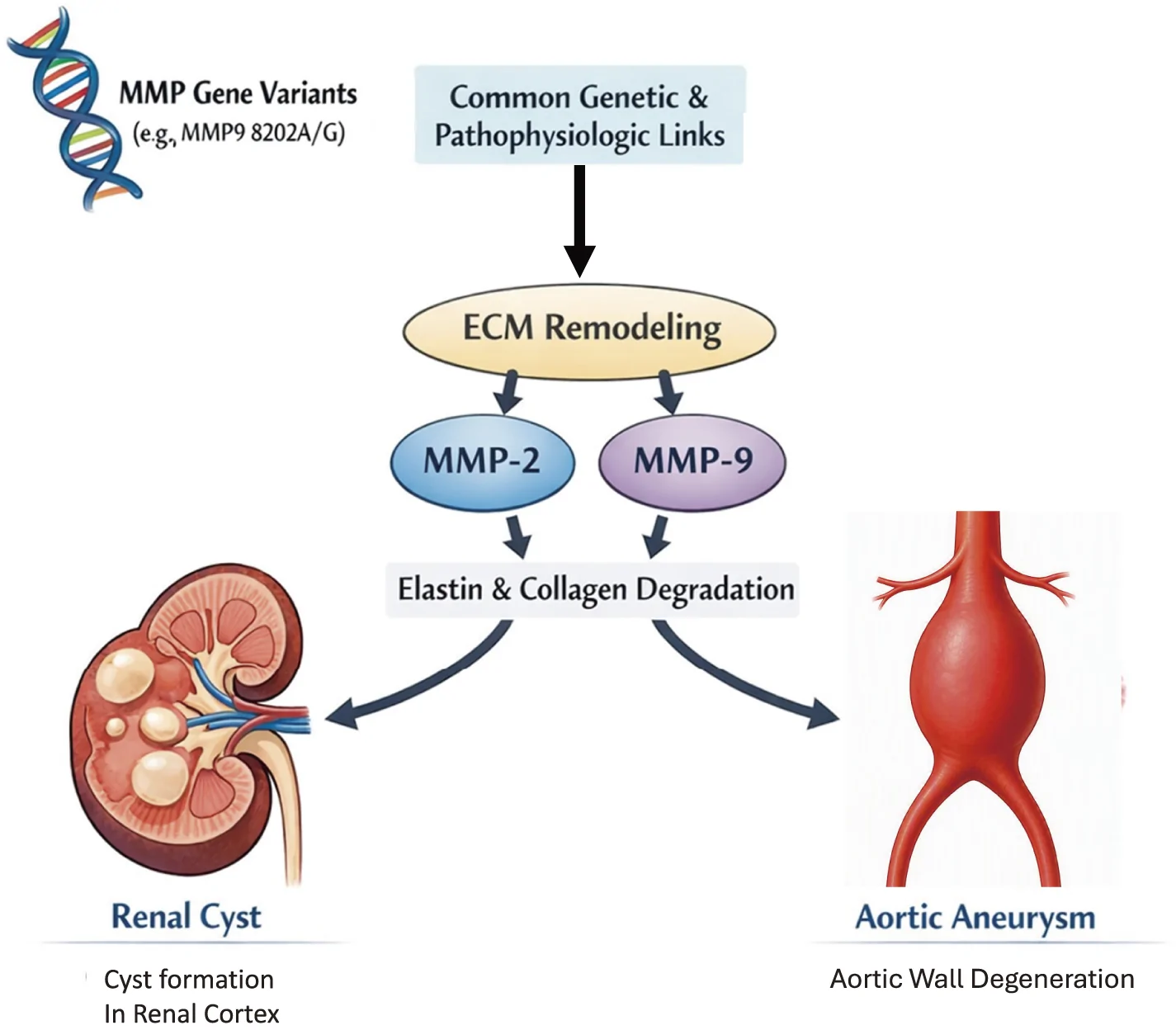
Proposed biological mechanisms linking renal cysts and aortic aneurysms. Shared genetic and pathophysiologic factors, including MMP gene variants and connective tissue disorders such as Marfan syndrome, may promote ECM remodeling. Upregulation of MMP-2 and MMP-9 leads to elastin and collagen degradation, contributing to cyst expansion in the renal cortex and medial degeneration of the aortic wall, ultimately resulting in renal cyst formation and aortic aneurysm formation. MMP, matrix metalloproteinase; ECM, extracellular matrix

The recurring association between SRCs and aortic aneurysmal disease suggests shared biological underpinnings. One potential pathway involves ECM remodeling, a fundamental process in aneurysm formation. Matrix metalloproteinases (MMP-2 and MMP-9) mediate elastin and collagen degradation, promoting both cyst expansion in the renal cortex and medial degeneration of the aortic wall.^28,29)^ Parallel elevations in MMP activity have been documented in aneurysmal tissue as well as in cyst fluid,^29,30)^ providing a plausible mechanistic bridge between renal and aortic pathology.

A second potential link is genetic susceptibility, with a shared genetic or pathophysiologic pathway between aortic aneurysms and SRCs having been proposed. Variants in MMP-related genes, including the 8202A/G polymorphism of MMP-9, may predispose individuals to both TAA and renal cyst formation.^31)^ This concept is further supported by findings in Marfan syndrome, where comparable ECM abnormalities underlie both aneurysmal and cystic changes.^4,27)^

Arterial aging and stiffness constitute a third, convergent mechanism. With advancing age, elastin fragmentation and collagen accumulation progressively reduce vascular compliance. We reported that, in a cohort of 223 AAA patients, the presence of SRCs was independently associated with increased arterial stiffness, defined as brachial–ankle pulse wave velocity ≥1800 cm/s, even after adjusting for age, blood pressure, and hypertension.^22)^ These findings support the interpretation of SRCs as markers of systemic vascular aging and advanced ECM deterioration.

Importantly, aneurysm formation and sac shrinkage after EVAR/TEVAR represent distinct biological processes. Aneurysm development is primarily driven by chronic inflammatory and proteolytic degradation of the ECM and progressive weakening of the aortic wall,^28,29)^ whereas postoperative sac remodeling is largely determined by procedural and local factors, including endoleak, endotension, intrasac thrombus organization, and the structural integrity of the aneurysm wall.^32,33)^ Despite these mechanistic differences, both processes appear to be influenced by systemic ECM degeneration and connective tissue fragility. Accordingly, SRCs may be interpreted as imaging markers of generalized ECM vulnerability that predispose patients to aneurysm formation and limit postoperative sac regression.

## Potential Prognostic Role of SRCs after Endovascular Aortic Repair

Current evidence is limited and is based primarily on retrospective observational studies. However, the available data consistently support an association between SRCs and postoperative aortic remodeling or complications after EVAR and TEVAR.^5–10)^ Although further validation in independent prospective cohorts is needed, SRCs represent a promising imaging marker for identifying patients at increased risk of impaired postoperative sac remodeling.

### SRCs and aneurysm sac shrinkage after EVAR (Table 3A)

**Table 3:** Role of simple renal cysts (SRCs) as prognostic indicators after endovascular aortic repair

A. Sac shrinkage after endovascular aneurysm repair (EVAR) for abdominal aortic aneurysm (AAA)			
First author (Year)	Cases (n)	SRC prevalence (%)	Sac shrinkage (with SRCs vs. without SRCs)
Nishibe (2020)^5)^	155	59.0%	1 yr: 19.2% vs 42.4%, P = .003/2 yr: 19.6% vs 53.3%, P = 0.001
Kano (2022)^6)^	143	60.1%	1 yr: 42% vs 69%, P = 0.002
B. Sac shrinkage after thoracic endovascular aortic repair (TEVAR) for degenerative thoracic aortic aneurysm (TAA)			
First author (Year)	Cases (n)	SRC prevalence (%)	Sac shrinkage (with SRCs vs. without SRCs)
Kano (2023)^7)^	103	44.6%	1 yr: 23.9% vs 59.6%, P <0.001
C. Aortic-related adverse events (ARAEs) after TEVAR for type B aortic dissection (AD)			
First author (Year)	Cases (n)	SRC prevalence (%)	ARAEs or distal aortic negative remodeling
Lu (2019)^8)^	238	43.7%	24-mo ARAEs: Overall HR 1.962 (95% CI 1.023–3.764, P = 0.043)/Chronic dissection HR 8.841 (95% CI 1.726–45.294, P = 0.009)
Zhu (2022)^9)^	428 (matched cohort)	50%	5-yr ARAEs: HR 1.84 (95%CI 1.13–3.00, P = 0.014)
Wang (2026)^10)^	209 (training set)	9.03%	2-yr distal aortic negative remodeling: OR 4.80 (95% CI: 1.32–17.52, P <0.05)

HR, hazard ratio; OR, odds ratio; CI, confidence interval

Our previous EVAR cohort of 155 patients, including 92 (59.0%) with SRCs and 63 (41.0%) without SRCs, demonstrated that SRCs were associated with attenuated aneurysm sac regression.^5)^ Patients with SRCs exhibited significantly lower rates of sac shrinkage at both 1 and 2 years after EVAR (19.2% vs 42.4% and 19.6% vs 53.3%, respectively). In multivariable analysis, SRCs remained an independent negative predictor of sac shrinkage (OR 0.28; 95% CI 0.12–0.63), whereas larger initial sac diameter was positively associated with sac shrinkage. Another EVAR cohort of 143 patients, including 86 (60.1%) with SRCs, which partially overlapped with the initial cohort and shared a subset of patients, again showed that renal cysts independently predicted impaired sac regression (OR 0.31; 95% CI 0.15–0.67).^6)^

Although type II endoleak was included as a covariate in our EVAR analysis, it was not identified as an independent predictor of sac shrinkage failure. Given that endoleak remains an important determinant of sac behavior,^31)^ further studies with comprehensive adjustment for established predictors are warranted to clarify the independent prognostic value of SRCs.

Taken together, these findings suggest that SRCs may represent a possible marker of unfavorable sac remodeling after EVAR. Although further validation in larger, independent cohorts with comprehensive adjustment for established predictors is required, SRCs may provide additional information for individualized risk stratification and postoperative surveillance.

### SRCs and aortic remodeling after TEVAR for degenerative TAA (Table 3B)

A similar pattern is evident in patients with degenerative TAA treated with TEVAR.^7)^ We found that 45% (46/103) of patients had SRCs, and the presence of SRCs was associated with markedly reduced sac shrinkage at 1 year. Sac regression occurred in 23.9% of patients with SRCs, compared with 59.6% of those without SRCs (P <0.001). The presence of SRCs remained an independent negative predictor of sac shrinkage (OR 0.15; 95% CI 0.06–0.40; P <0.001), whereas a larger baseline sac diameter independently favored postoperative regression (OR 1.08 per mm; 95% CI 1.03–1.14; P = 0.002). These findings suggest that the remodeling phenotype associated with SRCs extends from the abdominal to the thoracic aorta, suggesting that an SRC-linked systemic connective tissue disorder may manifest as impaired postoperative sac remodeling following TEVAR.

### SRCs and long-term aortic events after TEVAR for type B AD (Table 3C)

The prognostic relevance of SRCs is not confined to aneurysmal disease; rather, their influence extends to AD, where they independently predict aortic-related adverse events (ARAEs) or distal aortic negative remodeling following TEVAR.^8–10)^ ARAEs were defined as aortic-related mortality, new AD, aortic rupture, repeat TEVAR, paraplegia, major stroke, and other related complications.

Lu et al. reported that, in 238 hypertensive type B AD patients, SRCs were the only independent predictor of 24-month ARAEs (HR 1.96, 95% CI 1.02–3.76). In chronic dissection, the effect was even more pronounced, with SRCs showing a hazard ratio of 8.84 (95% CI 1.73–45.29), although this wide CI suggests limited precision due to the small number of chronic-phase cases and events.^8)^ Zhu et al. analyzed 718 TEVAR patients and showed that SRCs independently increased long-term ARAEs with a hazard ratio of 1.84 (95% CI 1.13–3.00) and raised rupture risk (HR 3.13, 95% CI 1.01–9.72).^9)^ Five-year cumulative incidence was consistently higher in patients with SRCs: ARAEs 28.9% vs 18.2%, and rupture 5.9% vs 2.1%. Wang et al. also found SRCs to be a strong independent predictor of distal aortic negative remodeling after TEVAR, with an odds ratio of 4.80 (95% CI 1.32–17.52).^10)^ Even when incorporating inflammatory indices (systemic inflammatory response index, SIRI; systemic inflammation index, SII; prognostic nutritional index, PNI), distal tears, and false lumen patency, SRCs remained one of the most powerful clinical predictors of false lumen expansion and true lumen collapse.

These data indicate that SRCs are not a benign incidental finding but a reproducible clinical marker of progressive aortic disease, associated with higher rates of adverse remodeling, persistent false lumen perfusion, and late complications after TEVAR.

## Integrative Perspective and Clinical Implications

The available evidence suggests that SRCs are more than incidental age-related renal findings. They may represent an imaging marker of systemic ECM degeneration and connective tissue vulnerability, key biological processes underlying the development and progression of AAA, TAA, and AD.

Clinically, SRCs are readily identified on routine imaging and may provide additional information for risk assessment.^34)^ Emerging evidence also suggests a potential role for SRCs in predicting postoperative outcomes after EVAR and TEVAR. However, current evidence is derived predominantly from retrospective observational studies and is insufficient to support changes in clinical management. Future multicenter prospective studies and mechanistic investigations are needed to validate these associations and establish the clinical value of SRCs for risk stratification and outcome prediction.

### Study limitations

Several priorities for future research emerge. First, most evidence is based on retrospective observational studies, highlighting the need for well-designed, ideally multicenter, cohort studies to determine whether SRCs are independent predictors of incident aortic disease, aneurysm growth, and postoperative outcomes beyond age and conventional risk factors. Second, mechanistic studies integrating renal and aortic tissue, linking MMP expression patterns, elastin and collagen architecture, and fibrillin integrity, may help clarify causal pathways and identify therapeutic targets. Third, advanced imaging approaches, such as radiomics, 4D-flow MRI, and elastography, could quantify wall mechanics and microstructure, thereby refining risk stratification in patients with SRCs. Fourth, integrated risk models that incorporate SRC status with demographics, comorbidities, pulse wave velocity, and aneurysm morphology may improve predictions of sac behavior and late events after EVAR/TEVAR. Finally, interventional research is warranted to evaluate whether targeted control of arterial stiffness or ECM-modulating strategies can offset the adverse postoperative profiles observed in patients with SRCs.

## Conclusions

Although the traditional view of SRCs as benign incidental findings has evolved, current evidence indicates that SRCs are associated with AAA, TAA, and AD, likely reflecting systemic ECM degeneration rather than isolated renal aging. Recent studies further demonstrate that SRCs carry prognostic relevance after EVAR and TEVAR, being linked to reduced sac shrinkage and, in specific cohorts, higher rates of long-term ARAEs.

Recognizing SRC status during diagnostic work-up, preoperative counseling, and postoperative follow-up may therefore improve individual risk assessment in patients with, or at risk for, aortic disease. As mechanistic and prospective data continue to develop, SRCs may become incorporated into risk models that guide imaging intervals, adjunctive assessment, and potentially device choice or targeted medical therapy.
